# Giant biatrial myxoma nearly obstructing the orifice of the inferior vena cava

**DOI:** 10.1186/1749-8090-8-148

**Published:** 2013-06-10

**Authors:** Chang-Hu Choi, Chul-Hyun Park, Ji-Sung Kim, Yang-Bin Jeon, Jae-Ik Lee, Kook-Yang Park

**Affiliations:** 1 Department of Thoracic and Cardiovascular Surgery, Gachon University Gil Hospital, Incheon, Republic of Korea

**Keywords:** Heart neoplasm, Myxoma, Biatrial

## Abstract

Cardiac myxomas are the most common type of benign cardiac tumors and most of them occur in the left atrium but the biatrial myxoma is uncommon. We present a rare case of giant biatrial myxoma nearly obstructing the orifice of the inferior vena cava. A 58-year old woman presented with exertional dyspnea and intermittent chest discomfort. The non-pedunculated tumor involved most of the interatrial septum and extended from the orifice of the inferior vena cava to the displaced mitral annulus and the lower left pulmonary vein. The resected specimen weighed 76 gram and measured 80 × 40 × 30 mm. She did not complain of dyspnea or show any sign of recurrence by echocardiography during the 2-year follow-up period.

## Background

Cardiac myxoma is the most common type of primary cardiac neoplasm and accounts for 30% to 50% of all primary tumors of the heart with an annual incidence of 0.5 per million populations [1,2]. Over 70% of all cardiac myxomas originate from the left and 18% from the right atrium. Biatrial myxomas account for less than 2.5% of all cardiac myxomas [3-5]. We present biatrial myxoma, which occupies both atria without pedunculating mass.

## Case presentation

**Figure 1:**
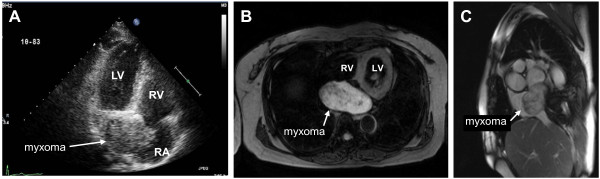
The large tumor, occupying both atria, was shown in the transthoracic echocardiography (A) and the magnetic resonance scan (B,C).

**Figure 2:**
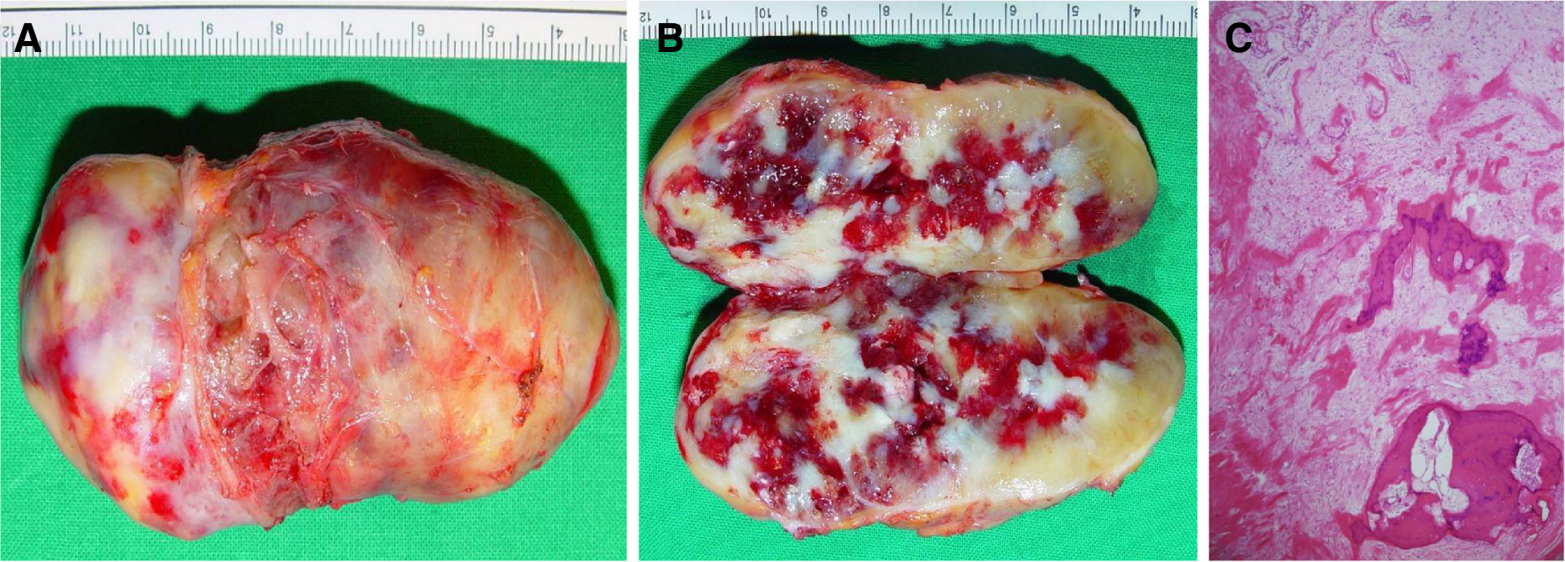
The myxoma was not gelatinous but solid (A) and showed the multi-focal calcification and hemorrhage (B,C).

A 58-year old woman presented with exertional dyspnea and intermittent chest discomfort. She had no past medical or familial history, and physical and neurological examinations, chest radiography, and electrocardiography findings were normal, although ESR was high at 54 mm/hr. However, echocardiography revealed a huge, immobile mass without pedunculation occupying both atria (Figure 1A). Due to its atypical characteristics and position, we performed a magnetic resonance imaging scan. Images showed a well-defined mass within the inferior portions of both atria with high signal intensity on T2 weighted images, slightly high signal intensity on T1 weighted images, no fat suppression on fat saturated images, and strong peripheral enhancement on gadolinium enhanced images. In addition, multiple calcified lesions of low intensity were noted within the tumor and the coronary sinus was inferiorly displaced by the mass (Figure 1B,C). Surgery was performed via conventional median sternotomy. During cardiopulmonary bypass, IVC cannulation was performed through the left femoral vein instead of the lower right atrium because the inferior vena cava was almost totally obstructed and the distal embolization should be prevented. The tumor was resected and the interatrial septal defect was closed with pericardium. The non-pedunculated tumor involved most of the interatrial septum and extended from the orifice of the inferior vena cava to the displaced mitral annulus and the lower left pulmonary vein. The resected specimen weighed 76 gram and measured 80 × 40 × 30 mm and was not gelatinous but solid. The one third of the right side of the specimen protruded into the right atrium, covered with endothelium but the two third of the left side of the specimen was attached to the base of the left atrium. The cut surface showed the multi-focal calcification and hemorrhage. Histologic examination confirmed myxoma without any other malignancy (Figure 2A,B,C). The patient was discharged without any complications, and during the 2-year follow-up period, she did not complain of dyspnea or show any sign of recurrence by echocardiography.

## Discussion

Cardiac myxoma is usually polypoid and pedunculated lesions, arising from endocardium [5]. In contrast, other cardiac tumors, such as, lipoma and rhabdomyoma are typically not pedunculated. The majority of cardiac myxomas manifest as a gelatinous, smooth, round mass with a glistening surface [1]. Imperio et al. reported that biatrial myxomas had two stalks arising from the same area of the interatrial septum and growing in opposite directions toward the right and left atria [6]. Irani et al. reported that there was no evidence of extension across the interatrial septum, thereby favoring contemporaneous origin of two separate tumors [3], whereas Ha et al. described two distinct types of myxoma, that is, a round type, characterized by a solid texture and round shape with a non-mobile surface (52%); and a polypoid type, characterized by a soft texture and an irregular shape with a mobile surface (48%) [7]. Right-sided myxomas are rare, but typically attach on a broad base and are more likely to be calcified than left-sided lesions [8,9]. In our case, the biatrial myxoma was not pedunculated, was attached on a broad base, and its surface was hard and solid with calcifications. We speculated that the myxoma grew to nearly obstruct the orifice of the IVC and that its root grow predominantly toward the left-side. This speculation proved consistent with surgical findings, as two thirds of the left-side of the specimen was attached to the base of the left atrium. The symptom of right side heart failure might have been erroneous because the IVC was not entirely occluded. Budd-Chiari syndrome can rarely develop by hepatic inferior vena cava and portal vein thrombosis, caused by a large myxoma of the right atrium [10]. The incidence of biatrial myxoma is rare but cardiopulmonary problems and neurologic complications can be prevented by surgical resection. Although the rate of recurrence is less than 5% after complete resection, [11] patients should be monitored routinely by cardiac echocardiography.

## Conclusion

The Biatrial myxoma is uncommon and could be considered in the differential diagnosis of other cardiac tumors with several imaging modalities. Prognosis is excellent after surgical resection*.*

## Consent

Written and informed consent obtained for publication of this case report and accompanying images. A copy of the written consent is available for review by editor-in- chief of this journal.

## Competing interests

The authors declare that they have no competing interests.

## Authors’ contributions

CP and CC collected the data and wrote the manuscript. JK, YJ, JL, and KP participated in the design of the manuscript and they revised and critically reviewed the manuscript. All authors read and approved the final manuscript.
